# Ultra-distal bypass remains a valuable option for tibial disease with tissue loss

**DOI:** 10.1016/j.jvscit.2026.102157

**Published:** 2026-01-24

**Authors:** Waad Ahmed, Megan Power Foley, Seamus McHugh, Sayed Aly, Peter Naughton, Daragh Moneley, Elrasheid Kheirelseid

**Affiliations:** a Vascular Surgery Department, Beaumont Hospital, Dublin, Ireland; b Surgery Division, Royal College of Surgeons Ireland, Dublin, Ireland; c General Surgery Speciality, Sudan Medical Specialization Board, Khartoum, Sudan

**Keywords:** Chronic kidney disease, Diabetic, Distal bypass, Patency, Wound

## Abstract

**Background:**

The pattern of distal tibial calcific disease associated with chronic kidney disease (CKD) and diabetes is challenging to revascularize. Ultra-distal bypass, on to pedal or plantar vessels, is an option for patients who have failed an endovascular approach. The goal of such ultra-distal bypasses is often to achieve wound healing rather than long-term patency. We present a retrospective observational study from an Irish tertiary vascular center.

**Methods:**

A retrospective review of all ultra-distal bypasses since 2019 was performed. Cases were identified from theater logs. Electronic health records and formal charts were used to record demographics, comorbidities and outcomes. Statistical analysis was performed using SPSS.

**Results:**

Since 2019, 25 limbs in 23 patients underwent ultra-distal bypass for tissue loss, 16 to pedal vessels and 9 to plantars. All patients were male and the median age was 62.0 years (range, 43-85 years). Seventy-two percent (n = 18/25) of the cohort were diabetic, 36% had chronic kidney disease, 4 (16%) were dialysis dependent, and 5 (20%) had congestive heart failure. Median follow-up duration was 14 months (range, 0-51 months). At 12 months, freedom from graft failure was 68% (n = 17/25) and freedom from major amputation was 92% (n = 23/25). At the most recent clinic review, 85% (n = 17/20) of ulcers had healed.

**Conclusions:**

Ultra-distal bypasses are a valuable tool in a vascular surgeon's arsenal for tissue loss in patients with complex tibial disease. Although the durability of bypass was variable, the majority of patients included in this study healed their ulcers and only three limbs progressed to major amputation.

**Article Highlights**

• **Type of Research:** Multicenter retrospective case series
• **Key Findings:** Twenty-five ultradistal bypasses in patients with tibial disease and tissue loss resulted in 80% wound healing. To date, three patients underwent below-knee amputations yielding a limb salvage rate of 88%. There was no difference in outcomes between patients with and without diabetes. All-cause mortality was 20%.
• **Take Home Message:** Despite the moderate graft patency rates in 3 years, limb salvage rate was high. This highlights that patency of graft is not necessarily needed for wound healing.

Peripheral artery disease (PAD) currently affects 230 million adults worldwide and is expected to increase with the aging population and rising incidence of diabetes mellitus.^1^ Chronic limb-threatening ischemia (CLTI), the most advanced stage of PAD, is a complex disease state associated with a high risk of major amputation and cardiovascular mortality.^2^ An aggressive approach to limb salvage in suitable patients can preserve individual patients' independence and quality of life, as well as decrease overall health care costs.^3^ In particular, diabetic patients are significantly more likely to develop gangrene and have a 10-fold higher risk of limb loss compared with nondiabetic PAD patients.^4^^,^^5^ Similarly, patients with chronic kidney disease (CKD), itself a common sequelae of diabetes, are at increased risk of limb loss that progressively worsens with ascending CKD stage.^6^ The synergistic effect of diabetes and renal impairment creates a specific histological pattern of calcified and high resistance tibial and malleolar vessels.^7^ This creates a particular challenge for revascularization, through either an endovascular or open approach.^8^^,^^9^ The small caliber (<1 mm) of pedal target vessels leads to significant size disparity with any conduit, coupled with limited distance for run-off into the pedal arch, creating a low-flow velocity system prone to thrombosis.

With the recent significant technical advances in the minimally invasive treatment of tibial disease, the number of endovascular revascularizations performed in contemporary practice far outweighs open surgery; however, pedal bypass remains a valuable tool for vascular surgeons.^10^, ^11^, ^12^, ^13^, ^14^ Although high-quality evidence is lacking, several recent retrospective comparative studies comparing tibial angioplasty to distal bypass in patients with infragenicular disease noted higher limb salvage rates in the open revascularization group at the cost of higher perioperative medical complication rates and longer hospital stays^6^^,^^15^, ^16^, ^17^ Despite a lack of high-quality evidence, the 2019 Society for Vascular Surgery CLTI guidelines preferentially suggested open bypass for patients with more complex lesions (Global Limb Anatomic Staging System 3-4) and severe tissue loss (Wound, Ischemia, and foot Infection 3-4).^18^ For such patients, the aim of any intervention focuses on wound healing and limb salvage, rather than long-term patency. Although some of the largest case series focusing on pedal and plantar bypasses predate the modern endovascular era, they report limb salvage rates of 70% to 87% at 3 to 4 years.^19^, ^20^, ^21^, ^22^, ^23^

The aim of this study was not to compare bypass to endovascular techniques for tibial lesions, but to provide insights into the role of ultra-distal bypasses in managing complex cases in comorbid patients in the endovascular era. This contemporary retrospective observational study examines all ultra-distal bypasses performed by a single consultant vascular surgeon in an Irish tertiary vascular center with a colocated dialysis unit. The primary outcomes were wound healing and limb salvage at 1 and 3 years, and secondary outcomes included primary patency, secondary patency, and amputation-free survival.

## Methods

Audit approval was granted by the institutional audit committee (CA2025/204R). The study was reported according to the STROBE guidelines for observational studies.^24^ A single-center retrospective review of all ultra-distal bypasses performed in a university-affiliated vascular department colocated with the national kidney transplant service between 2019 and 2024 was conducted. Our institutional policy is an endovascular-first approach for tibial disease, with exceptions made on a case-by-case basis for to preserve potential bypass outflow targets. All cases were performed by a single consultant vascular surgeon with a special interest in para- and inframalleolar bypass. Cases were identified from operating theater logs and a departmental prospectively maintained database. Inclusion criteria included all bypasses where the outflow anastomosis was at the ankle level or distal. A comprehensive review of patient charts and clinic letters were used to gather data on patient demographics, comorbidities, surgical details, adjunct procedures, complications, and length of stay for index admission, as well as subsequent follow-up, wound progress, and reinterventions. This process included the retrieval of both electronic records and archived paper charts, with manual extraction of operative details, laboratory data, imaging findings, and follow-up documentation for each patient individually. For the purpose of recording, CKD was defined as stage >3 CKD (estimate glomerular filtration rate of <60) and congestive heart failure was proven on echocardiogram or invasive cardiac testing. Preoperative and postoperative imaging was identified from the National Integrated Medical Imaging System records. As was standard in our institution, all patients underwent conventional angiography before bypass to identify a distal target vessel.

Patients were followed through December 31, 2024. The primary outcomes of interest were overall rates of wound healing and limb salvage. The secondary outcomes were primary patency, secondary patency, and amputation-free survival. The impact of diabetes and dialysis-dependent renal failure on outcomes were analyzed. The overall patency rate and wound healing results were estimated using surveillance duplex imaging and clinical examination findings during outpatient follow-up. For the purposes of this study, a bypass was defined as patent if there was a documented palpable pulse in the bypass or distally on clinic review by a senior vascular surgeon or if confirmed via surveillance imaging, such as a graft duplex or angiography.

All data were analyzed using SPSS Version 29.0 (IBM). Normally distributed continuous data were expressed as mean ± standard deviation, and the median (range) was used to describe the abnormally distributed continuous data. Categorical variables were presented as count and percent. Time intervals were characterized using median (range). Considering the small sample size, Fisher exact tests were used to analyze categorical variables. The Mann-Whitney *U* test and Kruskal-Wallis test were used to analyze nonparametric data. Kaplan-Meier survival curves were used to assess overall patency and freedom from amputation at specific time points, censoring patients who died during follow-up and those who were lost to follow-up. The level of statistical significance for null hypothesis testing was a *P* value of <.05.

## Results

### Cohort demographics and comorbidities

Over 5 years, the vascular department performed a total of 25 ultra-distal bypasses on 25 limbs in 23 patients. The median age at index procedure was 62.0 years (range, 43-85 years) and all patients were male. As shown in Table I, the majority of patients were diabetic (n = 18 [72%]) and more than one-third had at least stage 3 CKD (n = 9 [36%]). Almost all patients were anemic preoperatively (hemoglobin < 13.5 g/dL) and 40% were hypoalbuminemic. All limbs had TransAtlantic Inter-Society Consensus C or D disease.^25^ All limbs had tissue loss at the time of surgery, including 13 nonhealing ulcers and 12 gangrenous wounds. The median total length of stay was 28 days (range, 12-89 days) with a median stay of 16 days (range, 6-68 days) from index procedure to discharge.

**Table I:** Perioperative characteristics of cohort

	No.	%
Demographics and comorbidities		
Male sex	25	100
Age >70 years	11	44
Diabetes	18	72
CKD	9	36
Dialysis	4	16
Hypertension	22	88
Dyslipidemia	25	100
Ischemic heart disease	10	40
Previous myocardial infarction	9	36
Congestive heart failure	5	20
Chronic obstructive pulmonary disease	1	4
Smoking history (n = 14)	11	78.5
Indication for intervention		
Presenting symptoms		
Tissue loss	24	96
Rest pain	18	72
Chronic osteomyelitis	1	4
Perioperative biochemical parameters, median (range)		
Hemoglobin, g/dL	10.6 (7.6-14.3)	
Hemoglobin A1c mmol/mol, (n = 12)	48.5 (34-85)	
Albumin, g/L	34.0 (21-47)	
Creatinine, μmol/L	88.0 (44-542)	

*CKD,* Chronic kidney disease.

Postoperative medications are outlined in Supplementary Table I (online only). All patients were discharged on a statin and an antiplatelet. Aspirin monotherapy was prescribed in 84% of cases (n = 21/25), two patient received clopidogrel monotherapy and two patients were on both agents as dual antiplatelet therapy. Aside from the two patients on dual antiplatelet therapy, 19 patients were placed on full-dose anticoagulation and antiplatelet monotherapy.

### Technical surgical details

Surgical details are outlined in Table II. Before undergoing distal bypass, 13 limbs (52%) underwent attempted endovascular revascularization with an ipsilateral angioplasty, the majority of which targeted tibial vessels. Bypass inflow was taken from the superficial femoral artery in six cases and popliteal artery in 19 cases. Outflow vessels were pedal (n = 16) or plantar (n = 8); one limb had an outflow to a tarsal artery. Ipsilateral single-segment GSV was used as the conduit in 96% of limbs, whereas in one limb a composite graft of GSV and synthetic graft was used. Seven limbs underwent adjunct outflow procedures. These procedures included outflow endarterectomy (n = 2/7) and arch angioplasties (n = 5/7).

**Table II:** Surgical details

	No.	%
Preoperative endovascular procedure		
Ipsilateral digital subtraction angiogram	25	100
Foot arch complete (n = 21)	3	14.25
Previous ipsilateral angioplasty	13	52
SFA-popliteal	3	
Tibial	11	
Bypass inflow		
SFA	6	24
Popliteal	19	76
Bypass outflow		
DPA	8	32
PTA	8	32
Medial plantar	3	12
Lateral plantar	5	20
Tarsal	1	4
Bypass conduit		
SS GSV	24	96
Composite (GSV and synthetic)	1	4
Adjunct procedures		
Outflow endarterectomy	2	8
Arch angioplasty	5	20
Ipsilateral toe amputation		
During index procedure	2	8
During index admission	11	44
Complications		
Wound infection	0	
Wound dehiscence	1	4
Thrombosis (early)	4	16
Hematoma	2	8

*DPA,* Dorsalis pedis artery; *GSV,* great saphenous vein; *PTA,* posterior tibial artery; *SFA,* superficial femoral artery; *SS GSV,* single segment great saphenous vein.

Circumferential dissection of the distal target vessel was avoided; instead, only the minimum length of the artery required to control the vessel and create an arteriotomy was used. Vessel control of the proximal and distal ends was achieved using vessel loops rather than tourniquet control, and an Esmarch bandage was not used in this cohort. The intraoperative flow measurement was assessed with a completion angiogram. Postoperatively, duplex ultrasound surveillance was used to assess the patency, in addition to the clinical correlations. Skin closure was performed in a tension-free manner, with selective use of adjunctive wound management strategies rather than routine pie crusting. Two limbs underwent ipsilateral digital amputation and debridement of necrotic tissues at the time of the index procedure and a further 11 had subsequent digital amputations during the index admission. Immediate postoperative complications included graft thrombosis in four limbs requiring embolectomy, two minor wound hematomas, and wound dehiscence at the distal anastomosis managed with negative pressure wound therapy.

### Wound care adjuncts

Wound management was individualized based on wound characteristics and infection status and included surgical debridement where indicated, negative pressure wound therapy, and advanced dressings such as Aquacel or Iodoflex. Postoperatively, wounds were examined regularly, and wound care strategies were adjusted in conjunction with multidisciplinary input to support healing once inline flow had been restored.

### Follow-up and reintervention rates

At December 31, 2024, median follow-up for all limbs was 19.0 months (range, 0-63.5.0 months), with 72% of cases (n = 18) having a least 12 months of follow-up post index procedure. Among the patients with the seven limbs with <12 months follow-up, two died and two underwent major amputations within 12 months of surgery. To date, 12 individual limbs (48%) have required reinterventions, with one limb requiring two reinterventions on the same segment (Supplementary Table II, online only). Early reintervention was required for seven limbs at a median of 3 days postindex procedure (range, 0-8 days) and six limbs had a late reintervention at a median of 4 months postindex procedure (range, 2-37 months). Types of reinterventions included graft thrombectomy, revision of distal anastomosis with endarterectomy and vein patch, catheter-directed thrombolysis, and angioplasty.

### Wound healing, graft patency, limb salvage, and amputation-free survival

**Fig 1:**
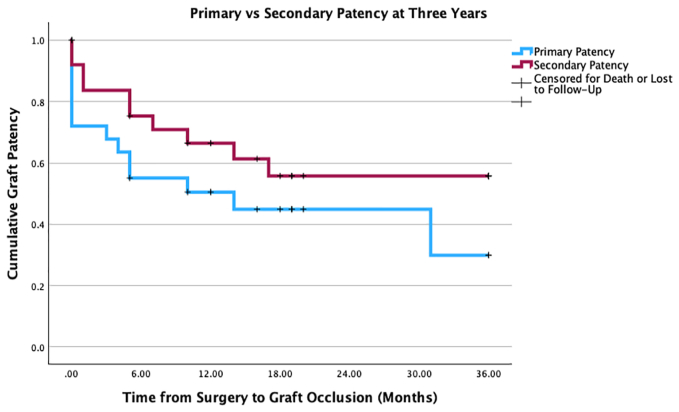
Primary and secondary patency of the bypasses at 3 years. This Kaplan-Meier curve compares the primary and secondary patency of the bypasses. This is shown at 1, 2, and 3 years.

**Fig 2:**
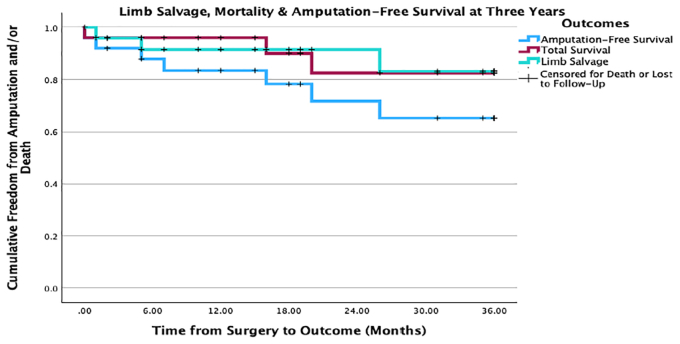
Amputation-free survival, total survival, and limb salvage rates at 3 years. This Kaplan-Meier curves compares the total survival, amputation-free survival, and limb salvage rates of the cohort at 1, 2, and 3 years. It is shown in the figure where part of the cohort is censored for death or lost to follow-up.

At the last clinic review, 80% of wounds (n = 20/25) had healed, including either the initial tissue loss present at index procedure or subsequent digital amputation sites. Of the five wounds recorded as unhealed at the end of study period, one patient had died with an unhealed wound in the immediate postperiod period, one patient was lost to follow-up, two patients underwent below-knee amputations for progressive ischemia, and one remains under active surveillance. At the end of the study period, allowing for different durations of follow-up, 56% of grafts (n = 14/11) were patent. The primary and secondary patency rates were 52% (n = 13/25) and 68% (n = 17/25) at 1 year, and 44% (n = 11/25) and 60% (n = 15/25) at 3 years, respectively (Fig 1). To date, three limbs have required major amputation, with an overall 3-year limb salvage rate of 88% (n = 22/25) (Fig 2).

Two amputations were performed shortly after graft occlusion owing to progressive ischemia; the third was performed for a new wound that developed 25 months after the graft was shown to be occluded. There was no significant difference in limb salvage rates between either diabetics and nondiabetics (83.3% vs 100%; *P* = .534) or dialysis and nondialysis patients (100% vs 86%; *P* = 1.000). By the end of the study period, the all-cause mortality rate was 20% (n = 5/25). The amputation-free survival rates at 1 and 3 years were 84% (n = 21/25) and 72% (n = 18/25), respectively (Fig 2).

The impact of comorbidities and technical aspects on overall patency (combined primary and secondary) and wound healing at 3 years are outlined in Table III. Significantly fewer patients who needed either an arch angioplasty for an incomplete foot arch at index procedure or a reintervention at any time had a patent graft at the end of follow-up (*P* = .005 and *P* = .015, respectively). A greater proportion of diabetic patients had occluded grafts compared with nondiabetic patients, although it did not reach significance (50% vs 14%; *P* = .179). Of the four dialysis patients, two had patent grafts at last review and two were occluded (*P* = 1.00). Correspondingly, proportionally more dialysis patients had nonhealed wounds, although this difference did not reach significance (40% vs 10%; *P* = .166). All three limbs that ultimately required major amputation had an occluded graft, which trended toward significance (*P* = .052). Similarly, proportionally more nonhealing wounds were in patients with occluded grafts, although it did not reach significance (80% vs 20%; *P* = .121). Similar to patency rates, proportionally more patients who needed adjunct arch angioplasty at the index procedure or a subsequent reintervention did not have healed wounds at 3 years.

**Table III:** Association between patient variables and outcomes at 3 years

Factor	Overall patency			Wound healing		
	Patent (n = 15)	Nonpatent (n = 10)	*P* value	Healed (n = 20)	Unhealed (n = 5)	*P* value
Patient factors						
Age >70 years	8/15 (53.3)	6/10 (60)	1.000	11/20 (55)	3/5 (60)	1.000
Diabetic	9/15 (60)	9/10 (90)	.179	14/20 (70)	4/5 (80)	1.000
CKD	5/15 (33.3)	4/10 (40)	1.000	6/20 (30)	3/5 (60)	.312
Dialysis	2/15 (13.3)	2/10 (20)	1.000	2/20 (10)	2/5 (40)	.166
IHD	6/15 (40)	4/10 (40)	1.000	9/20 (45)	1/5 (20)	.615
CCF	2/15 (13.3)	3/10 (30)	.358	4/20 (20)	1/5 (20)	1.000
HTN	13.15 (86.7)	9/10 (90)	1.000	17/20 (85)	5/5 (100)	1.000
Technical factors						
Anticoagulated	12/15 (80)	7/10 (70)	.653	16/20 (80)	3/5 (60)	.562
Glucophage	6/15 (40)	5/10 (50)	.697	9/20 (45)	2/5 (40)	1.000
Complete foot arch	3/15 (20)	0/6 (0)	.526	3/18 (16.7)	0/3 (0)	1.000
Preoperative angioplasty	6/15 (40)	7/10 (60)	.226	9/20 (45)	4/5 (80)	.322
Pedal outflow level	11/15 (73.3)	5/10 (50)	.397	13/20 (65)	3/5 (60)	1.000
Arch angioplasty	0/15 (0)	5/10 (50)	**.005**	3/20 (15)	2/5 (40)	.252
Reintervention at any time	4/15 (26.7)	8/10 (80)	**.015**	8/20 (40)	4/5 (80)	.160
Outcomes						
Wound healing	14/15 (93.3)	6/10 (60)	.121	-	-	-
Major amputation	0/15 (0)	3/10 (30)	.052	1/20 (5)	2/5 (40)	.091
Death	3/15 (20)	0/10 (0)	.250	2/20 (10)	1/5 (20)	.504

*CCF,* Congestive cardiac failure; *CKD,* chronic kidney disease; *HTN,* hypertension; *IHD,* ischemic heart disease.

Values are group size/cohort size (%).

Boldface entries indicate statistical significance.

## Discussion

This case series highlights that ultra-distal bypass in carefully selected patients still plays a significant role in the era of endovascular therapies. Over a 5-year period, a single consultant performed 25 ultra-distal bypasses for tissue loss. In the context of volume affecting outcomes of rare procedures, this annual number was comparable with other small volume case series.^26^, ^27^, ^28^ The primary and secondary patency rates at 1 and 3 years were 52% and 68% and 44% and 60%, respectively. However, despite moderate bypass longevity, the 3-year limb salvage rate was 88%, and 80% of distal extremity wounds were successfully healed at the most recent clinic review. In the context of an endovascular era, these findings support a treatment strategy in which ultra-distal bypass is viewed as a wound healing strategy, rather than to achieve long-term graft durability. The presence of diabetes and hemodialysis did not significantly impact graft patency and limb salvage rates; however, the study numbers were likely too small for a sensitive analysis. In this cohort, graft patency and limb salvage rates were not significantly affected by end-stage renal disease or diabetes, likely reflecting the small sample size. Nonetheless, these comorbidities remain key drivers in the pathophysiology underlying the need for an ultra-distal bypass approach. At 3 years, amputation-free survival and overall survival rates were 72% and 88%, respectively, highlighting that the patient cohort that requires ultra-distal bypass is typically comorbid and high risk. Notably, 44% of limbs (n = 11/25) underwent an attempted ipsilateral tibial angioplasty and all had a conventional angiography before proceeding with pedal bypass, demonstrating an institutional policy of selective endovascular-first approach for patients with complex tibial disease.^29^ Regardless, this study proves that ultra-distal bypass is reserved as an escalation plan to achieve adequate wound healing, in terms of failure of endovascular intervention.

### Comparison with other pedal bypass case series: Wound healing as the primary objective of ultra-distal bypass

These results were largely consistent with international studies, in particular the limb salvage rates. The most easily comparable study, also with small numbers of popliteal-pedal bypasses from another Irish center with a co-located dialysis unit, reported a 3-year secondary patency rate of 74.5%—higher than our 60.0%—and a limb salvage rate of 81.8%.^27^ In the largest case series with the longest median follow-up published to date, Pomposelli et al^19^ reported on 1032 dorsalis pedis bypasses with a 5-year secondary patency rate of 63% and a limb salvage rate of 78.2%. In a study of 98 plantar and tarsal bypasses, Hughes et al^28^ reported a 5-year secondary patency rate of 50% and limb salvage rate of 69%, although a large number of the cohort were lost to follow-up at this timepoint. In a large contemporary series of 208 limbs with tissue loss from Japan where more than one-half the cohort were dialysis-dependent, wound-healing rates at one year ranged from 79% to 84% (pedal and pedal branch), whereas primary and secondary patency rates were 53% and 73%, respectively.^30^ Across these multiple cohorts, ultra-distal bypass has shown its value as an option in achieving wound healing and limb salvage, in presence of high proportions of diabetes and dialysis-dependent patients. The persistent favorable gap between graft patency and limb salvage is interesting. Hughes et al^28^ previously postulated that some procedures may not have been necessary, that patients may not have had true CLTI. However, in most practice, an ultra-distal bypass represents a last resort technique reserved for nonhealing ulcers and gangrene, consistent with 78% to 100% rates of Rutherford V and VI CLTI in these studies. The authors strongly believe that primary objective of an ultra-distal bypass is to achieve limb salvage, rather than long-term patency, and that even a short duration of increased perfusion into the forefoot can sufficiently promote the healing process.^31^ In our cohort, of the 10 bypasses occluded at 3 years, 2 patients died with unhealed wounds, two limbs had progressive gangrene and required proximal amputation, three had already healed by the time the grafts occluded, and three limbs continued to successfully heal despite graft occlusion. This result was similar to Slim et al's findings,^32^ where at 1 year, 6 of 51 ultra-distal bypasses had occluded with one requiring a major amputation and four already had healed wounds.

### Impact of outflow, diabetes, and dialysis on outcomes

The numbers in this study were too small to facilitate a sensitive analysis of patient and technical factors that impacted graft patency and wound healing. We noted a trend of poorer graft patency in diabetic patients and a trend of poorer wound healing in dialysis patients, although neither were significant. In other studies with larger sample sizes, for both these comorbidities, the evidence supporting their impact—or lack thereof—on CLTI outcomes is conflicting, and the scope for interpatient heterogeneity among diabetics and dialysis patients is also wide and should be considered. In a series of 74 paramalleolar and inframalleolar bypasses, Abualhin et al^21^ reported a 2-year secondary patency rate of 38% and 3-year limb salvage rate of 80.5% and noted that insulin-dependent diabetes was a significant negative risk factor for both primary patency and limb salvage. Kodama et al,^30^ in their case series of 208 limbs, the need for dialysis emerged as the only significant clinical factor negatively impacting wound healing rates. Other studies have also reported significantly lower limb salvage and overall survival rates after revascularization in diabetic patients.^18^^,^^33^^,^^34^ It has been suggested that, owing to poor outcomes and high perioperative risks, it is imprudent to attempt revascularization in diabetic dialysis patients. We would disagree with this outlook, particularly because the incidence of diabetes increases exponentially along with its common complication of diabetic nephropathy, and vascular surgeons will find themselves treating more complex tibial disease in progressively younger patients. The median age in this cohort (62 years) was notably younger than those from the earlier case series (range, 66.7-73.0 years).^18^^,^^28^^,^^30^^,^^32^

The presence of significant disease in both the crural vessels and foot arch is one of the strongest predictors of poor outcomes in the diabetic dialysis cohorts. In our study, the only factors significantly associated with poorer patency were the need for either an adjunct pedal arch angioplasty at the time of index procedure or subsequent reintervention at any time, although this significance did not persist for wound healing. We hypothesized that both variables reflected the importance of adequate run-off, that is, a patent foot arch or adequate collateralization, to maintain sufficient flow velocities through the bypass, particularly when compounded by the inevitable size mismatch between venous conduit and pedal artery. Regarding wound healing, the presence of a complete plantar arch is positively associated with limb salvage, with the majority of studies coming from endovascular series comparing arch revascularization to angiosome targeting.^35^, ^36^, ^37^ Conventional wisdom would support that an intact arch is better than an incomplete one for tissue perfusion and wound healing. This result of tissue healing can be enhanced by maximizing the outflow at the index procedure, such as through an arch angioplasty, which can increase the chance of wound healing, regardless of the long-term graft patency. However, the data on the impact of a complete foot arch on graft patency after ultra-distal bypass are very limited. In a series of mostly crural bypasses (n = 31/167 inframalleolar bypasses), Rashid et al^32^ reported a similar rate of primary patency at 1 year between limbs with complete and no plantar arches (58.4% vs 63.8% in favor of incomplete arches; *P* = .516). Conversely, at 2 years, the wound healing rate significantly favored patients with complete arches (*P* = .026).^32^ As such, we suggest that all attempts should be made at the index procedure to maximize outflow through the pedal arch and its collaterals; for patients with incomplete arches in the presence of an adequate outflow target, its absence should not entirely dissuade surgeons from attempting a bypass.

### Strengths and limitations

The strength of this study was that it encapsulated a single surgeon's practice, ensuring a homogenous technical approach to inframalleolar bypass cases. The most significant limitations were the small sample size, which reduced the sensitivity of the statistical analysis, and the retrospective nature of data collection, which impacted the availability of certain variables. We were unable, for instance, to accurately estimate time from surgery to wound healing. Furthermore, a comparative analysis of either successful endovascular procedures performed for similar patterns of tibial disease during the study period, or for limb outcomes in patients deemed unsuitable for distal bypass, were outside the scope of this observational study. However, we feel that our approach was consistent with the Society for Vascular Surgery CLTI guidelines of choosing a bespoke revascularization plan based on patient risk factors, anatomical complexity, and severity of tissue loss.^18^ Similarly, the conflicting results produced by BEST-CLI (Best Endovascular vs. Best Surgical Therapy in Patients With Critical Limb Ischemia) and BASIL-2 (Bypass versus Angioplasty in Severe Ischaemia of the Leg-2), particularly their struggle to recruit eligible patients with disease suitable for either approach, further highlights the critical role of individualizing revascularization strategies.^38^, ^39^, ^40^

## Conclusions

Ultra-distal bypasses are a crucial technique in the vascular surgeon's toolkit for managing ulceration and gangrene in patients with complex tibial disease. These procedures provide a vital option when conventional revascularization methods are insufficient. Although the durability of the bypasses varied among patients, the study demonstrated encouraging outcomes. Most patients successfully healed their ulcers, highlighting the effectiveness of this intervention in promoting limb salvage. Notably, only three limbs progressed to major amputation, underscoring the potential of ultra-distal bypasses to preserve limb function and improve quality of life in this challenging patient population.

## Author contributions

Conception and design: WA, MF, SH, SA, PN, DM, EK

Analysis and interpretation: WA, MF, EK

Data collection: WA, MF, EK

Writing the article: WA, MF, EK

Critical revision of the article: WA, MF, SH, SA, PN, DM, EK

Final approval of the article: WA, MF, SH, SA, PN, DM, EK

Statistical analysis: WA, MF, EK

Obtained funding: Not applicable

Overall responsibility: WA

## Funding

None.

## Disclosures

None.
