# Supplementary material for: Ultra-distal bypass remains a valuable option for tibial disease with tissue loss
Source: J Vasc Surg Cases Innov Tech. 2026 Jan 24;12(3):102157. doi: 10.1016/j.jvscit.2026.102157 (PMC12954191; doi:10.1016/j.jvscit.2026.102157)
Supplement: Supplementary Tables [file mmc1.docx]

***Supplemental data:***

| ***Supplemental table (I): Post-Operative Medications (n=24)*** | | |
| --- | --- | --- |
| Medication at Discharge (n=25) | N | % |
| Statin | 25 | 100 |
| B-Blocker | 11 | 44 |
| *Anti-Thrombotic Therapy* | | |
| **Any Anti-Platelet**  Anti-Platelet Monotherapy   - Aspirin - Plavix   Dual Anti-Platelet Therapy (Aspirin & Plavix) | 25  23   - 21 - 2   2 | 100 |
| **Anticoagulation**  Anticoagulation & Anti-Platelet   - Apixiban - Warfarin - Edoxaban - LWMH   Anticoagulation only | 19  - 16  - 1  - 1  - 1  0 | 76 |
| *Diabetic Medication (n=18)* | | |
| Glucophage | 11 | 61 |
| SGLT2 inhibitor | 6 | 33.3 |
| Insulin | 11 | 61 |

| ***Supplemental table (II): Technical details and success of early and late reinterventions.*** | | | | |
| --- | --- | --- | --- | --- |
| **Early Reintervention (≤30 days of Index Procedure)** | | | | |
| *Reintervention* | *Days since Index Procedure* | *Technical Success* | *Patent at 24 Hours* | *Patent at 12 Months* |
| Graft thrombectomy | 1 | Y | Y | Y |
| Graft thrombectomy | 3 | Y | N | N |
| Graft thrombectomy and dissection of chronic scar tissue at tarsal tunnel | 1 | N | N | N/A |
| PTA to outflow vessel (ATA) | 2 | Y | Y | Y |
| Graft thrombectomy, endarterectomy and revision of distal anastomosis with vein patch | 4 | Y | Y | Y |
| Graft thrombectomy, revision of distal anastomosis with vein patch angioplasty and outflow angioplasty of foot arch | 3 | Y | N | N |
| Revision of distal anastomosis with outflow vessel endarterectomy and vein patch | 8 | Y | Y | N |
|  | | | | |
| **Late Reintervention (>30 days of Index Procedure)** | | | | |
| *Reintervention* | *Months since Index Procedure* | *Technical Success* | *Patent at 24 Hours* | *Patent at 12 Months* |
| PTA to outflow vessel (ATA) for threatened bypass* | 26 | Y | Y | Y |
| PTA to inflow vessel (Distal SFA) for threatened bypass | 2 | Y | Y | Y |
| Aspiration thrombectomy & PTA to inflow vessel (Popliteal) | 37 | N | N | N |
| Catheter-directed thrombolysis & PTA to inflow vessel (SFA) | 4 | Y | Y | N |
| PTA to inflow vessel (Distal SFA) and stent to outflow | 4 | Y | Y | N |
| Endarterectomy of outflow vessel, revision distal anastomosis and plantar angioplasty | 4 | N | N | N |
| **early and late reintervention* | | | | |
